# Identification and characterization of a potential strain for the production of polyhydroxyalkanoate from glycerol

**DOI:** 10.3389/fmicb.2024.1413120

**Published:** 2024-06-20

**Authors:** Mengheng Xue, Rong Huang, Wei Liu, Jian Cheng, Yuwan Liu, Jie Zhang, Limei Wang, Dingyu Liu, Huifeng Jiang

**Affiliations:** ^1^School of Life Science and Technology, Wuhan Polytechnic University, Wuhan, China; ^2^Tianjin Institute of Industrial Biotechnology, Chinese Academy of Sciences, Tianjin, China; ^3^National Center of Technology Innovation for Synthetic Biology, Tianjin, China; ^4^College of Biotechnology, Tianjin University of Science and Technology, Tianjin, China; ^5^School of Life Sciences, Division of Life Sciences and Medicine, University of Science and Technology of China, Hefei, China

**Keywords:** PHB, polyhydroxyalkanoates, Ralstonia sp., glycerol utilization, third-generation sequencing

## Abstract

While poly (3-hydroxybutyrate) (PHB) holds promise as a bioplastic, its commercial utilization has been hampered by the high cost of raw materials. However, glycerol emerges as a viable feedstock for PHB production, offering a sustainable production approach and substantial cost reduction potential. Glycerol stands out as a promising feedstock for PHB production, offering a pathway toward sustainable manufacturing and considerable cost savings. The identification and characterization of strains capable of converting glycerol into PHB represent a pivotal strategy in advancing PHB production research. In this study, we isolated a strain, *Ralstonia* sp. RRA (RRA). The strain exhibits remarkable proficiency in synthesizing PHB from glycerol. With glycerol as the carbon source, RRA achieved a specific growth rate of 0.19 h^−1^, attaining a PHB content of approximately 50% within 30 h. Through third-generation genome and transcriptome sequencing, we elucidated the genome composition and identified a total of eight genes (*glpR*, *glpD*, *glpS*, *glpT*, *glpP*, *glpQ*, *glpV*, and *glpK*) involved in the glycerol metabolism pathway. Leveraging these findings, the strain RRA demonstrates significant promise in producing PHB from low-cost renewable carbon sources.

## Introduction

Poly (3-hydroxybutyrate) (PHB), the most extensively studied member of the polyhydroxyalkanoates (PHAs), represents a class of biodegradable and biocompatible polyesters. PHB exhibits a diverse array of chemical structures, physical properties, and thermoplastic characteristics (Chen, 2009; Gao et al., 2011; Chen and Patel, 2012), rendering it highly promising for applications in biomedical, food, and environmental fields (Chen, 2009; Gao et al., 2011). Researchers have been actively engaged in advancing the commercialization of PHB (Chen and Patel, 2012; Chen and Hajnal, 2015). The cost of raw materials has emerged as a substantial constraint, representing over half of the total production expenses (Tyo et al., 2010; Yu et al., 2019). Over the past three decades, progress in metabolic engineering has facilitated the synthesis of PHB from a range of feedstock, including glycerol, fatty acids, industrial waste, and sugars (Wang et al., 2013; Chen and Jiang, 2018; Sirohi and Pandey, 2019; Li and Wilkins, 2020; Sirohi et al., 2020). Among these options, glycerol stands out as an exceptionally attractive substrate for PHB production, offering a high degree of reduction and cost-effectiveness (Koller et al., 2008; Cavalheiro et al., 2009).

Currently, a diverse array of potential PHB-producing strains has been documented, including *Ralstonia eutropha* (*Cupriavidus necator*) (Xu et al., 2010), *Aquabacterium* sp. (Westbrook et al., 2019), *Halomonas campaniensis* (Russmayer et al., 2019), *Rhodospirillum rubrum* (Zhang et al., 2022), *Pseudodonghicola xiamenensis* (Feng et al., 2023), *Rhodococcus* sp. (Yue et al., 2014), *Priestia megaterium* (Sznajder and Jendrossek, 2011), *Streptomyces* sp. (Mostafa et al., 2020), *Erythrobacter aquimaris* (Trakunjae et al., 2021), *Rhodospirillum rubrum* (Vázquez et al., 1996), *Burkholderia* spp. (Krishnan et al., 2017; Mostafa et al., 2020), *Pandorea* sp. (Narancic et al., 2016), and so on. Among these, strains capable of converting glycerol into PHB have been identified, with *Bacillus* sp. and *Cupriavidus* sp. being commonly reported. Additionally, it is worth noting that bacteria are not the only organisms demonstrating excellence in PHA production from glycerol; certain archaea, such as *Haloferax mediterranei* (Coutinho de Paula et al., 2019), also exhibit promising capabilities in this regard. Expanding the strain library for PHB production is paramount, especially for Gram-negative bacteria with thinner cell walls. Additionally, it is essential to explore wild-type strains with industrial potential and to strategically design and construct PHB cell factories.

This study aimed to isolate and characterize a potential strain capable of efficiently utilizing glycerol to produce PHB. Recently, we have isolated a strain from *R. eutropha* H16 cultures with an excellent ability to consume pure glycerol for growth and PHB accumulation. The characterization of this strain offers valuable insights and enhances our understanding of the PHB production potential within the *Ralstonia* genus. One of the significant advantages of the biodiesel boom has been the substantial decrease in the market price of glycerol (de Paula et al., 2017; Oliveira-Filho et al., 2021), which currently ranges from approximately $566.42 to 599.33 per ton. This approach presents a novel solution by connecting the utilization of the chemical by-product glycerol with the production of a high-value, low-cost product, PHB, thereby achieving cost-effectiveness. Furthermore, the versatility of the novel bacterium suggests the potential for genetic engineering to expand its capability to produce a diverse array of products.

## Materials and methods

### Genome sequencing and assembly

The RRA genome underwent sequencing at the Beijing Genomics Institute (BGI, Shenzhen, China) utilizing the PacBio Sequel II and DNBSEQ platforms. The PacBio platform employed four SMRT cells Zero-Mode Waveguide arrays for sequencing, generating a set of subreads. Subreads with a length of less than 1 kb were filtered out. The Canu program facilitated self-correction, resulting in draft genomic units—uncontested groups of fragments—utilizing a high-quality corrected circular consensus sequence subreads set. To enhance the accuracy of the genome sequences, GATK^1^ was employed for single-base corrections.

### Genome component prediction

Gene prediction on the RRA genome assembly was conducted using glimmer3,^2^ leveraging Hidden Markov models. Recognition of tRNA, rRNA, and sRNAs utilized tRNAscan-SE (Lowe and Eddy, 1997), RNAmmer, and the Rfam database. Tandem repeats were annotated via the Tandem Repeat Finder,^3^ with a selection of minisatellite and microsatellite DNA based on their repeat unit characteristics. Genomic island analysis was performed using the Genomic Island Suite of Tools (GIST),^4^ employing the IslandPath-DIOMB, SIGI-HMM, and IslandPicker methods. Prophage regions were predicted utilizing the PHAge Search Tool (PHAST) web server,^5^ while CRISPR loci were identified using CRISPRFinder.

### Gene annotation and protein classification

The optimal hits were identified through the Blast alignment tool for function annotation. Seven databases, namely KEGG (Kyoto Encyclopedia of Genes and Genomes), COG (Clusters of Orthologous Groups), NR (Non-Redundant Protein Database), Swiss-Prot (Darda et al., 2019), GO (Gene Ontology), TrEMBL, and EggNOG, were utilized for comprehensive function annotation. Additionally, four databases were employed for pathogenicity and drug resistance analysis. Virulence factors and resistance genes were discerned using the core dataset from VFDB (Virulence Factors of Pathogenic Bacteria) and ARDB (Antibiotic Resistance Genes Database), alongside the Carbohydrate-Active enZYmes Database (CAZy). Type III secretion system effector proteins were identified using EffectiveT3.

### Comparative genomics and phylogenetic analysis

Genomic comparisons were conducted utilizing MUMmer and BLAST, focusing on core/pan genes of RRA The strains TS, FC1138, and ATCC 49129 were grouped using CD-HIT, employing a 50% pairwise identity threshold and a 0.7 length difference cutoff in amino acids. Gene families were established from the genes of RRA, TS, FC1138, and ATCC 49129, integrating multiple software tools: protein sequence alignment via BLAST, redundancy elimination through Solar, and gene family clustering using Hcluster_sg software. Phylogenetic trees were constructed using TreeBeST with the Neighbor-Joining (NJ) method.

### Culture conditions

The strains were cultured for passaging in 2YT medium for liquid cultures and 2YT medium supplemented with 1.5% agarose for solid cultures. The 2YT medium was sourced from Solarbio, China. The composition of the mineral salts basic medium per liter includes 3.8 g of Na2HPO4, 2.65 g of KH_2_PO_4_, 1.5 g of NH_4_Cl, and 0.2 g of MgSO_4_, supplemented with a 1,000x nutrient solution. The pH was adjusted to 6.0 using either HCl or NaOH. The nutrient solution was formulated per liter as follows: 9.7 g of FeCl_3_, 7.8 g of CaCl_2_, 0.218 g of CoCl_2_·6H_2_O, 0.156 g of CuSO_4_·5H_2_O, 0.118 g of NiCl_3_·6H_2_O, and 0.105 g of CrCl_3_·6H_2_O. Gentamicin at a concentration of 10 μg/mL was used as the antibiotic. All chemically pure mineral salts were procured from Shanghai Yuanye Bio-Technology Co., Ltd., while gentamicin was obtained from Solarbio, China. The incubation conditions for RRA were set at 30°C and 220 rpm, while BW25113 was incubated at 37°C and 220 rpm.

### Screening of RRA strain

In our laboratory, we have preserved strains of *Ralstonia* sp. Recently, we discovered multiple strains of *Ralstonia* sp. within the *R. eutropha* H16 culture medium. To identify the strain exhibiting robust glycerol utilization, we employed an enrichment culture method. Specifically, we inoculated the *R. eutropha* H16 culture medium into 50 mL of 1% glycerol mineral salts medium supplemented with 10 ng/mL of gentamicin for cultivation. Once the OD_600_ of the culture reached 3.0, we subcultured a small aliquot of the culture into a fresh medium. Simultaneously, we streaked a portion of the bacterial solution onto agar plates. After several passages, only a single strain of *Ralstonia* sp. was observed on the plates, indicating successful isolation of the desired strain with strong specific glycerol utilization.

### Gene amplification and sequencing

PCR assays were conducted in 50 μL reaction volumes, comprising 1 μL (100 ng) of template genomic DNA, 2 μL (10 pmol) each of forward and reverse primers, 25 μL of 2× Phanta mix, and 20 μL of ddH_2_O. The amplification protocol commenced with an initial denaturation step at 94°C for 2 min, followed by 30 amplification cycles. Each cycle consisted of denaturation at 94°C for 1 min, annealing at 55°C for 1 min, and extension at 72°C for 1 min and 30 s. The PCR products were subjected to electrophoresis in a 1% agarose gel (Agarose MP, Roche Diagnostics) for 30 min at 130 V in TAE buffer. Following electrophoresis, the gel was stained with ethidium bromide and visualized under UV light using a gel documentation system (Imagestore, Ultra Violet Products, Cambridge). Photographs of the gel were taken for further analysis. A 200–10000 bp DNA ladder (Bioline) was incorporated into all gels to facilitate standardization and sizing of the amplified fragments. Subsequently, the amplified genes were purified utilizing the TIANquick Midi Purification Kit (TIANGEN, Beijing, China) following the manufacturer’s protocol. The purified amplicons were then sent for sequencing to GENEWIZI (Tianjin, China).

### Determination of growth curves

Initially, RRA was inoculated into a 2YT medium supplemented with 10 ng/mL of Gentamicin. Upon reaching the logarithmic growth phase, indicated by an OD_600_ range of 0.6–1.0, 40 mL of bacterial culture was harvested. The culture was then centrifuged at 4000 rpm for 10 min to pellet the cells. Following centrifugation, the cell pellet was washed twice with ddH_2_O. Subsequently, the pellet was diluted with ddH_2_O to achieve an OD_600_ of 0.4, facilitating ease of handling for subsequent procedures.

The 48 deep-well plates utilized in the fully automated microbial growth curve analyzer were loaded with 950 μL of four different initial cultures. These cultures included 10 g/L fructose, 10 g/L glycerol, 10 g/L glucose, and 10 g/L sodium acetate basic salt medium. Each culture was prepared with four biological replicates for the robustness and accuracy of the experimental results. To minimize liquid evaporation, ddH2O was added around the periphery of each well. Subsequently, 50 μL of bacterial solution was added to each culture to ensure that the initial OD_600_ concentration of all samples was 0.02. The inoculated 48-well plate was then placed onto the fully automated microbial growth curve analyzer MicroScreen (Gering, China). The analyzer was set to operate at 800 rpm and 30°C, with OD_600_ measurements taken every 60 min.

### Specific growth rate assay

The specific growth rate is measured by the automatic microbial growth curve analyzer to measure the growth curve, and then the growth rate is calculated according to the following steps (Zhang et al., 2020):

The specific growth rate for each time period was determined (*μ_i_*):


$$\mu_{i}=\frac{\ln N_{i+1}-\ln N_{i}}{t_{i+1}-t_{i}}$$

Among them, *N*_*i* + 1_ is the microbial biomass at the end of the time period, *N_i_* is the microbial biomass at the beginning of the same time period, *t*_*i* + 1_ is the end time of the time period, and *t_i_* is the start time of the same time period (Buschke et al., 2013).

The maximum growth rate for each time period is calculated (*μ_max_*):

The maximum growth rate can be obtained by comparing the growth rates of all time periods:


$$\mu_{max}=\max\left(\mu_{1},\mu_{2},\mu_{3},\ldots \ldots \mu_{i+1}\right)$$

The average specific growth rate was determined (*μ*):

The average growth rate can be calculated using the following formula to reduce the error:


$$\mu =\frac{\mu_{max-2}+\mu_{max-1}+\mu_{max}+\mu_{max+1}+\mu_{max+2}}{5}$$

The five growth rates before and after the maximum growth rate are shown as *μ_max_*_-2_ to *μ_max_*_+2_.

### TEM sample of RRA

RRA was inoculated into three different culture media: 50 mL of 1 g/L glycerol, 10 g/L of glycerol, and 10 g/L of glucose mineral salt medium. Each culture was initiated with an OD_600_ of 0.02. Three biological replicates were prepared for each group. Samples were periodically withdrawn from each culture to measure the OD_600_, allowing for monitoring of bacterial growth dynamics under varying nutritional conditions. Upon reaching the logarithmic growth phase, 40 mL of culture from the shakers was collected. Subsequently, the culture was subjected to centrifugation in a refrigerated high-speed centrifuge operating at 4°C and 4,000 rpm for 15 min. Following centrifugation, the resulting pellets were washed twice with ddH_2_O to eliminate any residual medium components. This process ensured the isolation of bacterial cells from the culture medium for further downstream applications. The sample processing protocol for electron microscopy is as follows:

1. Fixation: immerse the samples in 2.5% glutaraldehyde solution for 12 h at 4°C.

2. Dehydration: rinse the samples four times for 10 min each with 0.1 M phosphate buffer (PB). Treat the samples with 2% osmium fixative for 1.5 h. Rinse the samples twice for 10 min each with 0.1 M PB. Wash the samples twice for 10 min each with ddH_2_O. Submerge the samples in 50% ethanol for 10 min. Repeat the ethanol incubation steps with 70% ethanol and 90% ethanol for 10 min each. Prepare a 90% ethanol:90% acetone mixture (1:1) and immerse the samples for 10 min. Finally, immerse the samples in 90% acetone for 10 min.

3. Permeabilization at room temperature: treat the samples three times for 8 min each with 100% acetone. Incubate the samples in a 1:1 mixture of acetone and resin (0.5 mL) for 1 h. Repeat the incubation step with a 1:2 mixture of acetone and resin (0.5 mL) for 1 h, avoiding disruption of cells. Submerge the samples in a 1:3 mixture of acetone and resin (0.5 mL) overnight, without disrupting cells.

4. Embedding: apply resin (0.4 mL) to the samples and incubate at room temperature for 3 h, repeating this step twice. Dry the samples at 60°C for 48 h.

### Construction of *phaCAB* cluster expression plasmid

In this study, two types of plasmids were constructed for the expression of the *phaCAB* cluster:

1. pBBR1-*Phacab*-CE: this plasmid contains the RRA self-contained promoter, enabling constitutive expression of the *phaCAB* cluster.
2. pBBR1-*Phacab*-IE: this plasmid features an L-arabinose-inducible promoter, allowing for induced expression of the *phaCAB* cluster.

### Plasmid transformation by electroporation

Plasmids were transformed into *E. coli* BW25113 cells through the electroporation method, employing a Gene Micropulser (Bio-Rad, United States). Plasmid DNA was mixed with chilled *E. coli* BW25113 cells immediately before electroporation treatment. The cells were subjected to single electrical pulses at 1.8 kV, corresponding to field strengths of 18 kV/cm, with pulse lengths lasting 5 ms. This process facilitated the efficient uptake of plasmid DNA by the bacterial cells, enabling successful transformation.

Plasmids were introduced into RRA cells using a similar electroporation method as described earlier. RRA cells were subjected to single electrical pulses at 2.5 kV. Following electroporation, the transformed RRA cells were then incubated at 30°C for 1 h to allow for recovery and expression of the introduced plasmids. This process facilitated the successful transformation of plasmids into RRA cells.

### Cell dry weight and PHB content measurement

The bacterial cells were harvested by centrifugation at 6000 rpm for 10 min using a high-speed freezing centrifuge, specifically the Sorvall ST 16R. Following centrifugation, the cells were washed twice with distilled water to remove any residual medium or contaminants. Subsequently, the washed cells were quickly frozen in liquid nitrogen. The frozen cells were then subjected to lyophilization in a freeze-dryer maintained at −40°C until a constant weight was achieved. This process effectively removed moisture from the cells, resulting in the production of lyophilized bacterial samples.

Intracellular polyhydroxybutyrate (PHB) content was analyzed by methanolysis of lyophilized cells in chloroform. The resulting products, including PHB, were examined using gas chromatography (GC-2014, SHIMADZU, Japan) with flame ionization detection (FID) (Lee and Kim, 2015). PHB standards obtained from SHANGHAIZZBIO CO., LTD, were used for calibration. This method allowed for accurate quantification of PHB content in the samples.

## Results

### Third-generation sequencing and genomic analysis

The isolated strain exhibited accelerated growth when cultivated in a mineral salt medium (Hermann-Krauss et al., 2013) supplemented with glycerol compared to its growth in 2× yeast extract and tryptone medium with *R. eutropha* H16. This facilitated its isolation and enrichment. Subsequently, RRA underwent third-generation sequencing and genomic analysis. The genomic data were deposited in the GenBank database under accession number GCA_037023145.1. The genome of RRA spans 6,496,418 base pairs (bp), encompassing a total of 6,194 genes with a combined length of 5,653,998 bp. The average gene length is 912.83 bp, constituting 87.03% of the genome length. The GC content is measured at 63.94%. Of the 6,194 genes, 11 different database annotations were conducted, yielding the following results: VFDB: 408 genes (6.58%), ARDB: 30 genes (0.48%), CAZy: 191 genes (3.08%), IPR: 4,959 genes (80.06%), Swiss-Prot: 2,197 genes (35.46%), COG: 4,491 genes (72.5%), CARD: 4 genes (0.06%), GO: 3,584 genes (57.86%), KEGG: 3,551 genes (57.32%), NR: 5,971 genes (96.39%), and T3SS: 987 genes (15.93%). In total, 6,008 genes (96.99%) were annotated across these databases. Annotation results for the COG, GO, and KEGG databases are found in Supplementary Figure S1. A CRISPR sequence was identified by CRISPRFinder on plasmid 2, spanning from position 125,246 to 125,325, with a total length of 79 base pairs (bp) (Supplementary Sequence S1).

**Figure 1:**
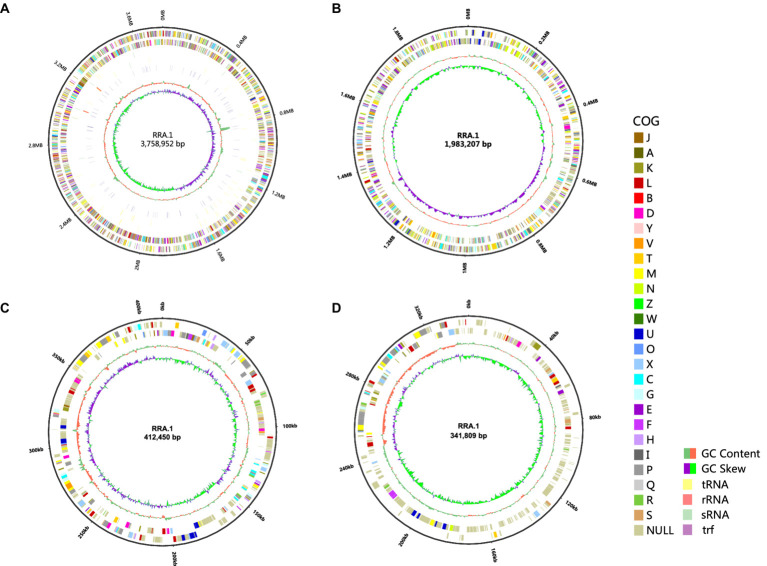
Genome circles of RRA **(A)** chromosome 1; **(B)** chromosome 2; **(C)** plasmid 1; **(D)** plasmid 2. From the outside to the inside, the circles represent the following information: genome size indicator, gene information on the forward and reverse strands (different colors of the coding sequences represent different COG functional categories), tRNA, rRNA, sRNA, trf, GC content, and GC skew (calculated as G-C/G + C). The size of the genome circular diagram is drawn in proportion to the size ratio of the chromosome and plasmids.

The entire genome of RRA (Figure 1) comprises 2 circular chromosomes one measuring 3,758,952 base pairs (bp) with a GC content of 63.39%, the other measuring 1,983,207 bp with a GC content of 63.79%. Additionally, it contains 2 circular plasmids: one measuring 412,450 bp with a GC content of 61.33% and the other measuring 341,809 bp with a GC content of 58.93%. The sequenced non-coding RNA (ncRNA) in RRA comprises 61 tRNA genes, 9 rRNA genes (comprising 3 5S rRNA genes, 3 16S rRNA genes, and 3 23S rRNA genes), and 20 sRNA genes. Furthermore, RRA harbors 183 tandem repeat sequences, totaling 13,889 base pairs (bp), which constitute 0.21% of the genome.

### 16s RNA sequence alignment and comparative genomics

**Figure 2:**
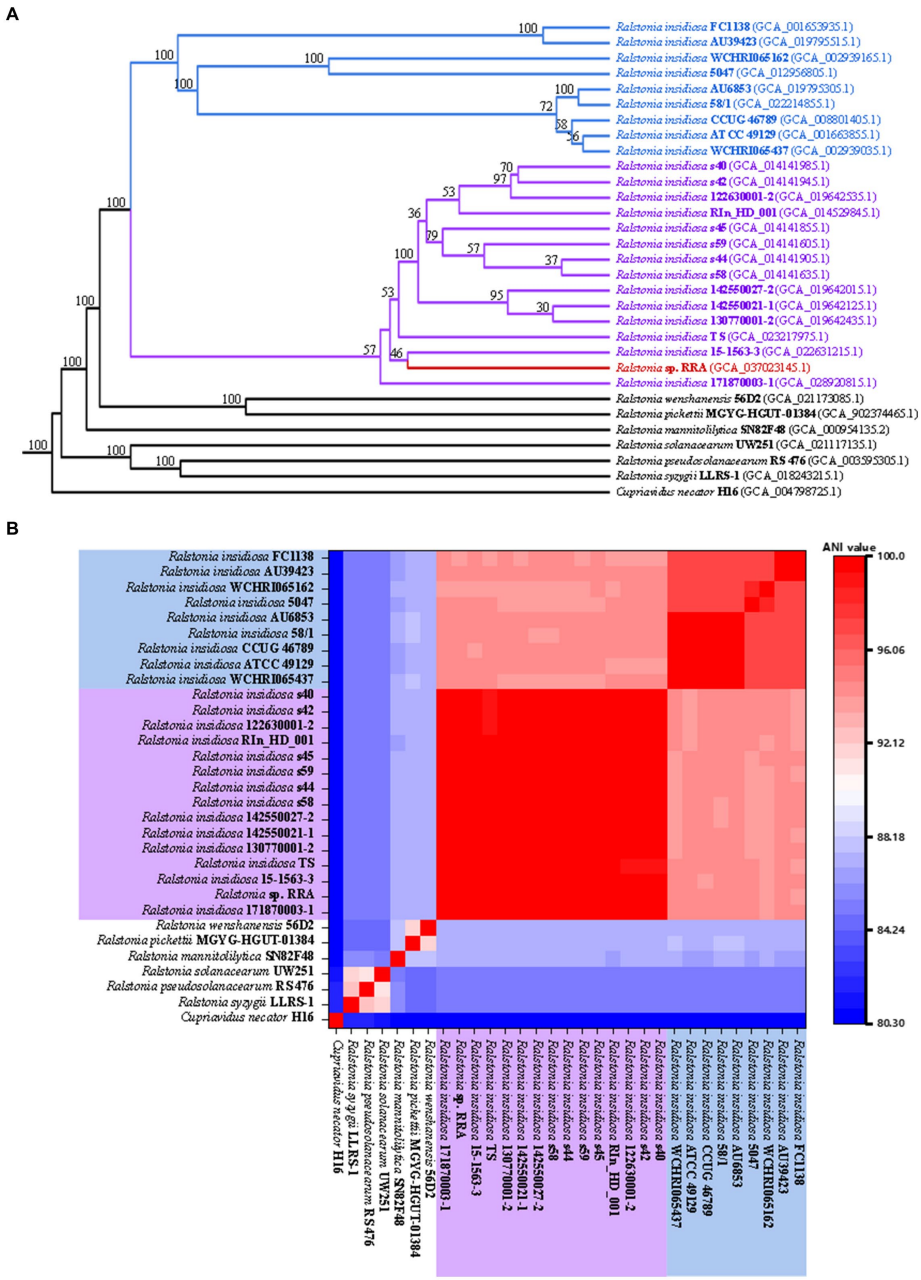
**(A)** Phylogenetic trees of RRA and other *Ralstonia* species. **(B)** The ANI comparison of RRA and other *Ralstonia* species. The species with blue and purple backgrounds correspond to the two branches of *Ralstonia insidiosa* in the phylogenetic tree.

During the strain identification process, we conducted a comparison of the 16S rRNA sequences, which yielded a fragment of 1523 base pairs (bp) for RRA. The BLAST results revealed that RRA is classified within the genus *Ralstonia* and exhibits a striking similarity of 99.79% to *Ralstonia insidiosa* ATCC 49129. Furthermore, we analyzed the distribution of shared orthologous clusters among *Ralstonia pickettii*, *R. eutropha*, RRA, and *Ralstonia insidiosa* ATCC 49129 using OrthoVenn2 (Yazdani and Gonzalez, 2007). The results of this analysis are depicted in Supplementary Figure S2. The analysis revealed that RRA and *Ralstonia insidiosa* ATCC 49129 share the largest number of orthologous clusters, totaling 795. However, it is important to note that while the 16S rRNA comparison provides initial insights, it may not be highly informative for species classification. Therefore, for a more precise determination of genus classification, modern methods such as average nucleotide identity (ANI) analysis and CDS similarity analysis are recommended. We utilized OrthoFinder (Johnson and Taconi, 2007) to identify 1379 single-copy gene families and constructed a phylogenetic tree using RAxML (Kato et al., 1996) for 31 strains (Figure 2A). This set included 6 *Ralstonia* species, 23 *R. insidiosa* strains, RRA, and the outgroup species *R. eutropha*. The analysis revealed that due to genetic diversity, the 23 *R. insidiosa* strains were segregated into two major branches during the evolutionary process. However, RRA and the representative strain *R. insidiosa* ATCC 49129 were positioned on separate branches, with *R. insidiosa* 15-1563-3 being the closest strain to RRA.

To validate the aforementioned inference, we computed the average nucleotide identity (ANI) between the 31 strains using FastANI software (Xu et al., 2019) and generated a heatmap (Figure 2B). ANI quantifies the similarity of homologous genes between two genomes, and it is commonly accepted that populations exhibiting close genetic relationships typically have ANI values of at least 70–75%, while strains with ANI values exceeding 95% are considered to belong to the same species (Stamatakis, 2014; Emms and Kelly, 2019). The clustering of the 31 genomes according to their ANI values mirrored the branching pattern observed in the phylogenetic tree. Additionally, the ANI value between RRA and strain ATCC 49129 was calculated at 94.3%.

**Figure 3:**
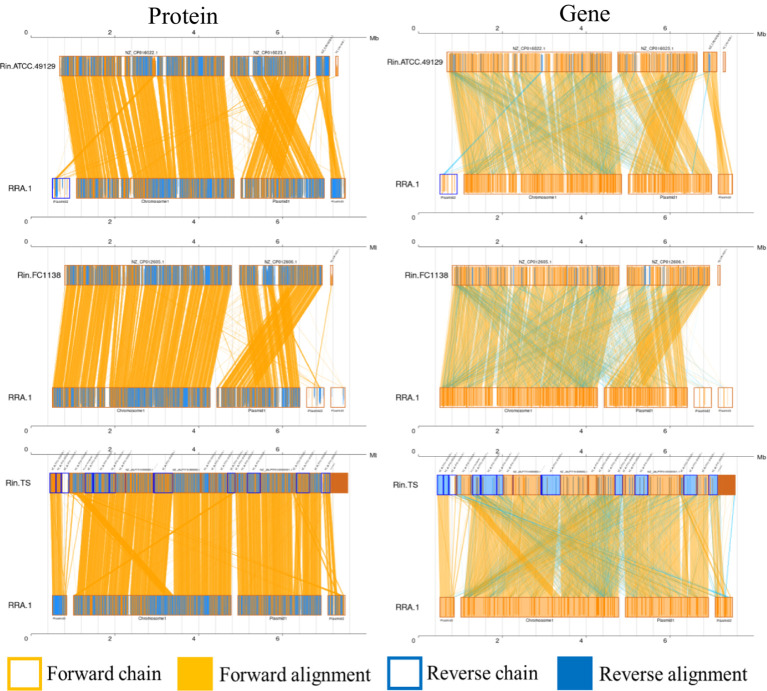
Co-linearity analysis diagram of RRA with ATCC 49129, FC1138, and TS, respectively. Collinear regions represent similar regions between two genomes. The orange boxes indicate that the directions of the two genomes are the same in a certain region, and the blue boxes indicate that the directions are opposite. The orange background indicates alignment between the forward strands of two genomes, and the blue background indicates alignment between the forward strands of one genome and the antisense strands of another genome.

Whole-genome alignment (WGA) entails the comparison of entire genomes between different species or strains. It examines how genes present in the genome of one species are arranged and positioned in the genome of another species. This comparison enables the analysis of genetic rearrangements, such as those caused by recombination and transposition events. WGA encompasses both nucleotide-level and amino acid-level comparisons, contrasting with analyses solely focused on amino acid sequences. Covariance analysis at the nucleotide level can unveil insights into insertions, deletions, and other sequence-related information. Figure 3 depicts the covariance between RRA and *Ralstonia insidiosa* strains TS, FC1138, and ATCC 49129, at both the nucleotide and amino acid levels.

### Characterization and analysis of transcriptome for strain RRA

**Figure 4:**
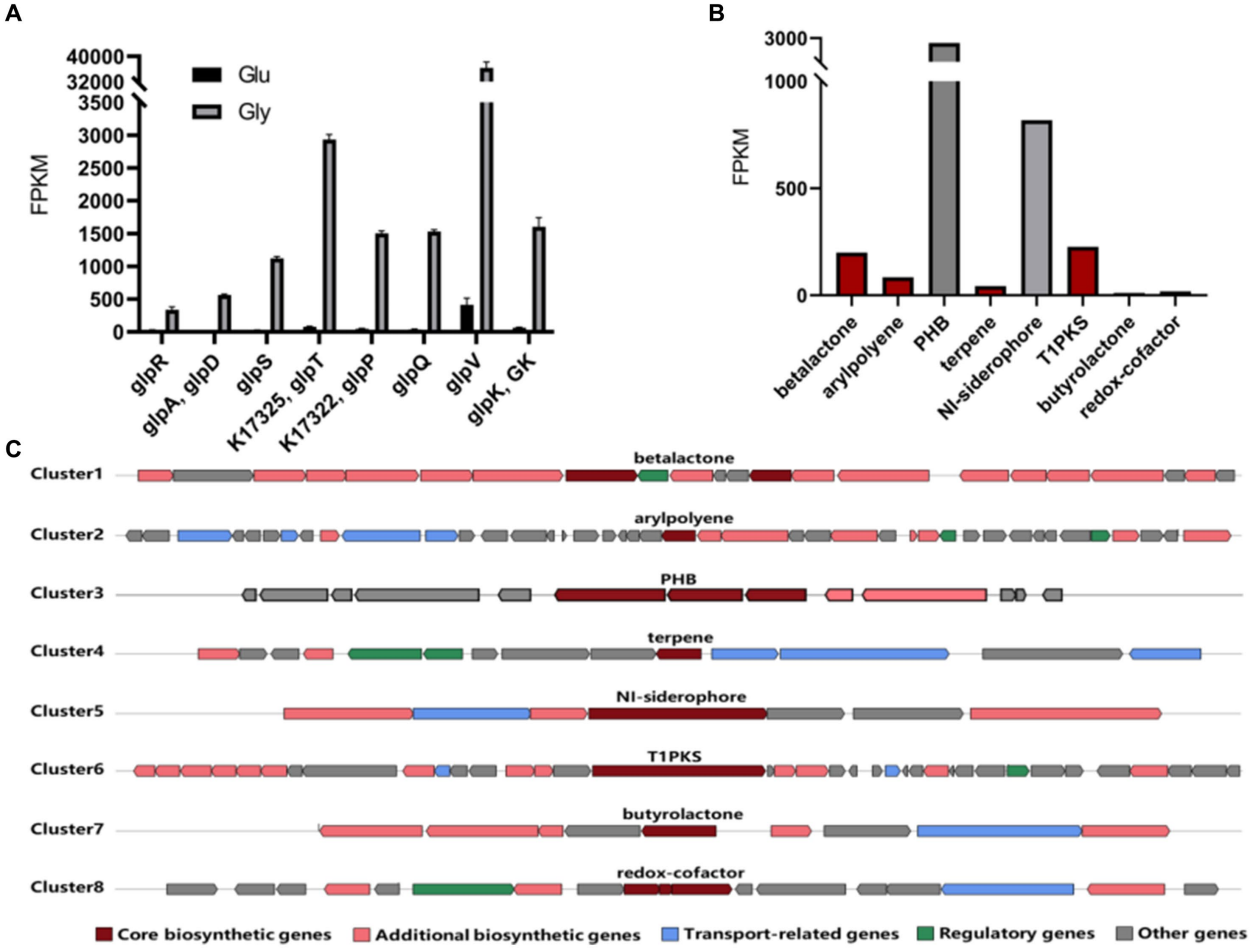
**(A)** Expression level of genes involved in the glycerol metabolism pathway in RRA. The black bars represent expression levels under glucose conditions, while the gray bars represent expression levels under glycerol conditions. **(B)** The FPKM values of key genes in the gene clusters of strain RRA when using glycerol as a carbon source. **(C)** Schematic diagram of predicted gene clusters in the genome of strain RRA.

To explore the mechanism of bioconversion under different carbon sources, we conducted a transcriptome analysis of strain RRA cultivated with 10 g/L of glycerol or glucose. The transcriptome data have been deposited in the Sequence Read Archive (SRA) database under accession number SRR28059441. Pearson’s correlation coefficient test and principal component analysis (PCA) affirm the accuracy and repeatability of the experimental methods. The transcript levels of a total of eight genes (*glpR*, *glpD*, *glpS*, *glpT*, *glpP*, *glpQ*, *glpV*, and *glpK*) exhibited significant changes in glycerol culture compared to glucose culture (Figure 4A). Among them, *glpD* and *glpK* are recognized as key genes in the glycerol metabolism pathway, encoding glycerol-3-phosphate dehydrogenase (GlpD) and glycerol kinase (GlpK), respectively. As for *glpR*, *glpS*, *glpT*, *glpP*, *glpQ*, and *glpV*, they encode the following proteins: DeoR/GlpR family DNA-binding transcription regulator (GlpR), ABC transporter ATP-binding protein (GlpS), ABC transporter ATP-binding protein (GlpT), sugar ABC transporter permease (GlpP), carbohydrate ABC transporter permease (GlpQ), and glycerol transport system permease protein (GlpV). Based on the FPKM (Fragments Per Kilobase of exon model per Million mapped fragments) (Jain et al., 2018) results, the expression levels of the glycerol metabolism cluster were notably elevated when glycerol was used as the carbon source compared to glucose, with an average ratio of 38:1. Specifically, genes such as *glpP* and *glpV* exhibited particularly pronounced increases in expression levels. Particularly noteworthy was the substantial difference in expression observed for *glpV*, which exhibited an 88-fold increase in expression when glycerol was utilized as the carbon source compared to glucose. This suggests that the efficient utilization of glycerol of RRA is likely attributed to its highly efficient glycerol transport system.

Moreover, we identified eight gene clusters responsible for the biosynthesis of high-value metabolites from the genome (Konstantinidis and Tiedje, 2005). Upon comparison with transcriptional data, it was observed that the metabolic fluxes of PHB and NI-siderophore were notably higher in RRA compared to other species (Figures 4B,C). After acquiring the PHB synthesizing genes of RRA, we conducted a comparison with those of *R. insidiosa* ATCC 49129 and *R. eutropha* (*C. necator*) H16 (Supplementary Figure S3).

### Growth analysis and PHB production of strain RRA

**Figure 5:**
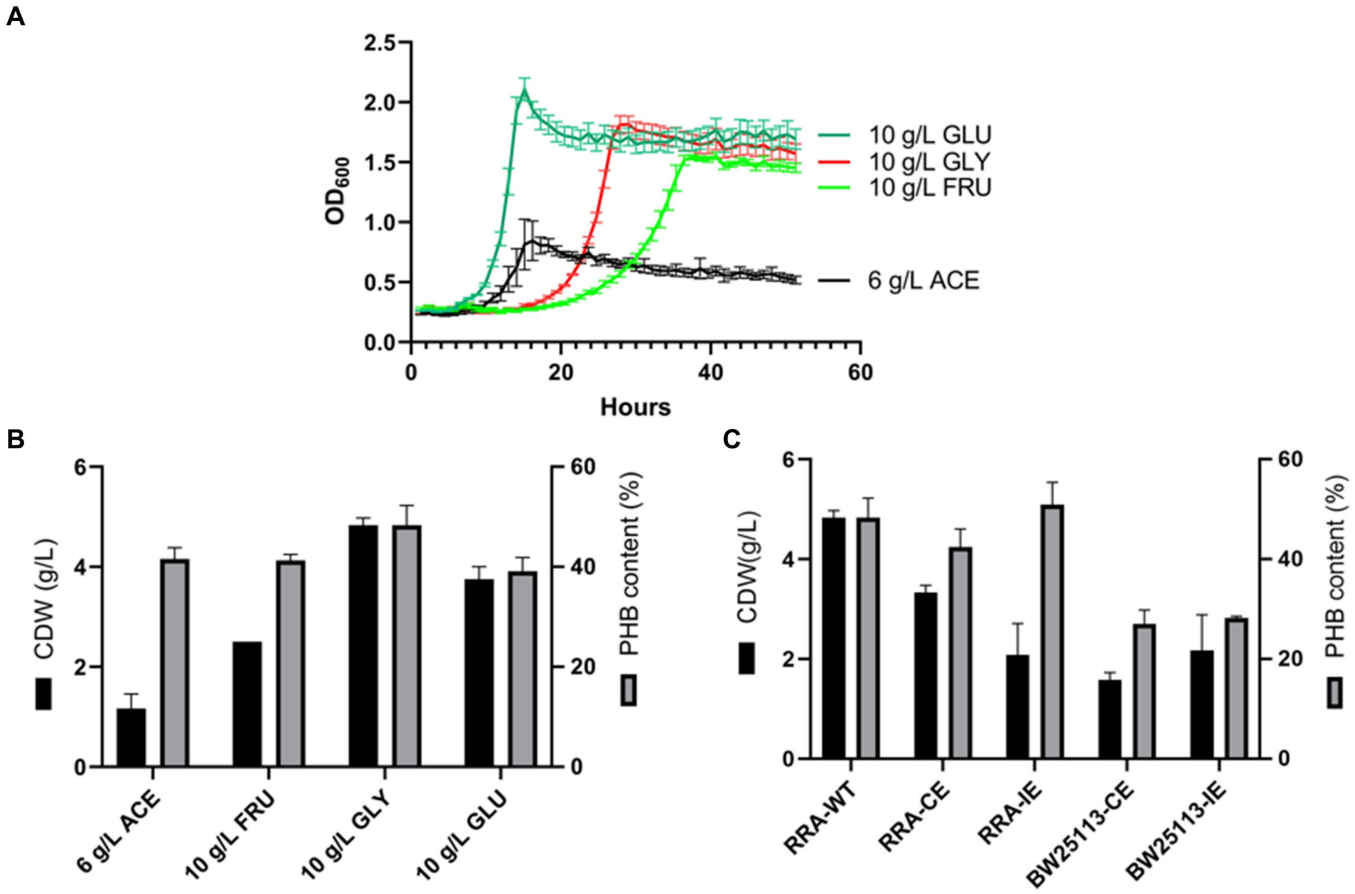
**(A)** Growth curves of RRA grew in glucose, glycerol, sodium acetate, and fructose. **(B)** PHB content and PHB output of RRA grew in glucose (10 g/L), fructose (10 g/L), glycerol (10 g/L), and sodium acetate (6 g/L) for 48 h. **(C)** PHB synthesis gene cluster expression under 1% glycerol in RRA and *Escherichia coli* BW25113, CE: pBBR1-*phaCAB*-CE with RRA self-contained promoter (constitutive expression), and IE: pBBR1-*phaCAB*-IE with L-arabinose-inducible promoter (induced expression).

The growth curves of strain RRA were assessed across various carbon sources, including glucose, fructose, glycerol, and sodium acetate (Figure 5A). When glycerol was utilized as the carbon source, the specific growth rate significantly increased, reaching 0.19 h^−1^ compared to 0.26 h^−1^ for glucose. To delve deeper into the mechanism of substrate metabolism in strain RRA, we conducted a transcriptome analysis of strain RRA cultivated with 1% (w/v) glycerol and glucose.

To assess the characterization of strain RRA for PHB production, the strain was cultured in shake flasks containing mineral medium supplemented with glucose, fructose, glycerol, and sodium acetate, respectively. The strain RRA yielded dry cell weights (DCW) of 4.49 g/L (with 2.25 g/L PHB) and 3.77 g/L (with 1.47 g/L PHB) after 30 h of cultivation in glycerol and glucose, respectively (Figure 5B). Clearly, significantly higher PHB accumulation occurred under the glycerol substrate compared to other substrates, reaching 50.1% of the dry cell weight (DCW), with a yield of 0.45 g/g. This elevated PHB productivity can be attributed to the efficient utilization of glycerol. To delve deeper into the function of the PHB synthesis gene cluster in strain RRA, we cloned the gene cluster onto the pBBR1 plasmid and subsequently transformed it into both RRA and *Escherichia coli* BW25113 (Figure 5C). The PHB gene cluster was expressed under both a constitutive promoter (the self-contained promoter on the *phaCAB* gene cluster of RRA) and an inducible promoter (L-arabinose-inducible expression), respectively. The PHB gene cluster was effectively expressed heterologously in *E. coli*, resulting in a maximum PHB content of 28% under various promoters. The recombinant RRA-CE strain exhibited enhanced PHB accumulation, with a 3% increase in dry cell weight (DCW) compared to the wild type, reaching up to 53.1%. However, the DCW of the bacteria was notably lower, at only 2.75 g/L.

### Phenotypic characterization

**Figure 6:**
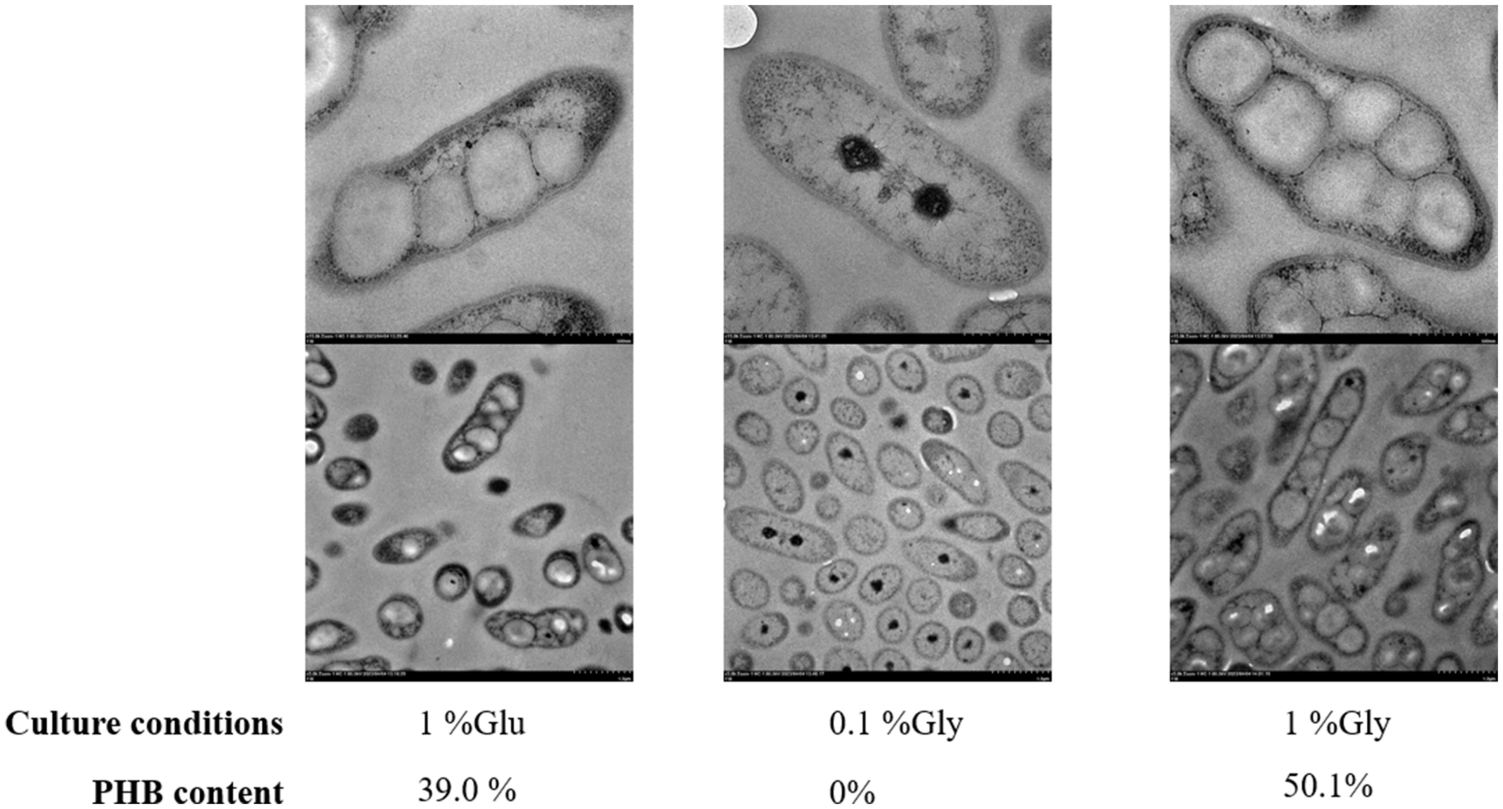
Transmission electron microscopy (TEM) of RRA grew in 1% glucose, 0.1% glycerol, and 1% glycerol for 48 h. The image bar at the top of each group is 500 nm and the one at the bottom is 1.0 μm.

The transmission electron microscopy (TEM) analysis of RRA under high glycerol, low glycerol, and high glucose concentrations was conducted as depicted in Figure 6. RRA exhibits an overall rod-shaped morphology, with both the cell wall and cell membrane clearly visible. In other PHB-producing strains, such as *R. eutropha*, PHB typically serves as a carbon reserve and stress protectant, accumulating in large quantities only when the external environment becomes unfavorable. For instance, under extreme gas autotrophic conditions, PHB can account for up to 90% of the dry weight of the bacterium. However, in the case of RRA, the PHB product induces a notable change in morphology, transforming the bacterium from a rod shape to a nearly spherical shape even under nutrient-rich conditions. In the TEM analysis of *R. insidiosa* of Nurjadi et al. (2020), none of the figures displayed PHB aggregation, and the overall cell morphology appeared to be ovoid, rather than the elongated rod-like shape observed in RRA.

## Discussion

In this study, the strain RRA was isolated and identified from *Cupriavidus necator* cultures with an excellent ability to utilize glycerol. The ANI analysis, whole genome alignment analysis, and evolutionary tree analysis were implemented to identify RRA as a branch of *Ralstonia* sp., and after analysis of its genome and transcriptome, we found that it has two potentially high-value products, PHB and NI-siderophore. Its native PHB synthesis gene could be heterologously expressed in *E. coli* by a constitutive or inducible promoter, allowing it to synthesize PHB. The increasing concern for energy and environmental sustainability has led to extensive research into improving green processes for converting sustainable resources into biochemicals (Mortazavi et al., 2008). Identifying the strain RRA has provided insight into the *Ralstonia* sp. and a new potential strain to produce PHB.

*Ralstonia* sp. has received a lot of attention in the last two decades for its excellent PHB production and has been used by many researchers as a model PHB-producing bacterium. *R. eutropha* was discovered and classified in Germany in the 1960s and was first designated as *Hydrogenomonas eutropha* due to its capability of utilizing molecular hydrogen and carbon dioxide as sole energy and carbon sources (Blin et al., 2023). The name tree has six items in four decades until *Cupriavidus necator* and *Wautersia eutropha* were considered as one species (Schlegel et al., 1961; Davis et al., 1969; Makkar and Casida, 1987; Kumar and Kim, 2018; Nurjadi et al., 2020) (Supplementary Figure S4). In this study, we observed distinctions between RRA and strain *R. insidiosa* based on both 16S rRNA sequences and ANI analysis, which suggests that RRA may represent a novel genus of bacteria. Nevertheless, these differences lie on the borderline of species classification. For instance, ANI analysis revealed a similarity of 94.3%, which is slightly below the customary 95% threshold for species classification. Nevertheless, the current limitations that prevent further phenotypic analyses of RRA and *R. insidiosa* mean that any reclassification of RRA as a new species is, at this stage, only speculative.

In recent times, the development of sustainable and environmentally friendly materials has attracted the attention of researchers as potential alternatives to petroleum-based materials (Yabuuchi et al., 1995; Vandamme and Coenye, 2004). Biodegradable microbial polyesters have been regarded as potential candidates to replace traditional petrochemical-derived plastics in a circular economy, especially in agricultural applications (Vaneechoutte et al., 2004; Sharma et al., 2023a). For instance, straw cellulose is a renewable and biodegradable material with the potential to be developed into cellulose nanofibers in agriculture. Such nanofibers could be used as a carrier for the delivery and release of fertilizer (Sharma et al., 2023b; Koshy et al., 2024). The recognition of such biomaterials as a solution to the problems of a sustainable, circular economy is becoming increasingly prevalent. At present, research on polyhydroxyalkanoates (PHAs) attracts more attention due to potentially wide applications based on their biocompatibility, biodegradability, and structural diversity (Kasirajan and Ngouajio, 2012). However, the production cost is still the greatest challenge hindering the expansion of the commercial PHB market (Tyo et al., 2010). The selection of feedstock plays a critical role in determining the economic feasibility and sustainability of the process. The production of PHAs from various biomass feedstocks such as glycerol, lignin, cellulose, and agro-industrial wastes has the potential to reduce the reliance on virgin materials and minimize waste, thereby enabling the establishment of a closed-loop system within a circular economy. Lignin is a particularly abundant renewable resource that has the potential to be utilized as a biomass feedstock for the synthesis of PHB (Sharma et al., 2023c). *R. eutropha* (*C. necator*) H16 was observed to accumulate biopolymer (PHA 0.6 g/L) using lignin derivatives as the sole source of carbon (Sharma et al., 2023d). The strain *R. eutropha* is capable of converting lignocellulose biomass to PHB. It can accumulate 11.4 g/L of PHB after 48 h of fermentation using rice straw as a feedstock (Moradali and Rehm, 2020). Glycerol-based waste materials are more readily processed and obtained than lignin and its derivatives. Crude glycerol is the chief by-product of biodiesel-producing industries, and the market price is low (Tomizawa et al., 2014; Chauhan et al., 2022). Hence, utilization of glycerol could provide a sustainable production model and significantly reduce the production cost of biopolymers. *Bacillus* sp. are capable of efficient production of PHB using glycerol or crude glycerol. The maximum PHB accumulation was obtained as 2.80 g/L using glycerol through *B. megaterium* (Saratale and Oh, 2015). *Bacillus* sp. ISTVK1 can accumulate 85.19% cell dry weight of PHA at optimized conditions using crude glycerol for the synthesis of PHA (Anitha et al., 2016). In this study, the RRA strain was able to accumulate 2.25 g/L of PHB in glycerol at 48 h, which exhibited excellent ability to convert glycerol to PHB, yielding 0.45 g/g glycerol. In addition, the strain RRA showed an excellent capacity for glycerol metabolism, and the specific growth rate reached 0.19 h^−1^ when using glycerol as the sole carbon source, significantly faster than that of *R. eutropha*. The wild-type *R. eutropha* was completely unable to grow in a medium with glycerol as the sole carbon source. Therefore, for the construction of engineered strains using glycerol as a substrate, the isolated wild-type RRA strain has greater potential. Furthermore, *Ralstonia* sp. should be expected to conduct genetic manipulation for the production of a wide range of products from glycerol.

The field of low-carbon resources used by microorganisms currently faces significant challenges (Gómez Cardozo et al., 2016; Mota et al., 2017). In particular, the adaptive evolution of substrate utilization combined with selection is a promising approach (Morya et al., 2018). However, isolation and identification of new strains are also efficient strategies that could reduce the time costs of strain evolution and modification (Grim et al., 2020). This study showed that the strain RRA has great potential for producing PHB from low-cost renewable carbon sources and reminded researchers of the importance of isolating new strains.

## Conclusion

In this study, we successfully identified and characterized a potential strain capable of efficiently utilizing glycerol to produce PHB. Through phylogenetic analyses, we provided valuable insights into *Ralstonia* sp. This research sheds light on the potential of this bacterial genus. The newfound strain exhibits significant promise for producing various products from low-cost renewable carbon sources.

## Data availability statement

The original contributions presented in the study are publicly available. The genomic data were deposited in the GenBank database under accession number GCA_037023145.1. Additional materials and data used and/or analyzed during the current study are available from the corresponding author on reasonable request.

## Author contributions

MX: Writing – original draft. RH: Writing – original draft. WL: Writing – original draft. JC: Writing – original draft, Writing – review & editing. YL: Writing – original draft, Writing – review & editing. JZ: Writing – original draft, Writing – review & editing. LW: Supervision, Writing – original draft. DL: Writing – original draft, Writing – review & editing. HJ: Writing – original draft, Writing – review & editing.

## Footnotes

^1^ https://www.broadinstitute.org/gatk/

^2^ http://www.cbcb.umd.edu/software/glimmer/

^3^ http://tandem.bu.edu/trf/trf.html

^4^ http://www5.esu.edu/cpsc/bioinfo/software/GIST/

^5^ http://phast.wishartlab.com/
