# Supplementary material for: Identification and characterization of a potential strain for the production of polyhydroxyalkanoate from glycerol
Source: Front Microbiol. 2024 Jun 20;15:1413120. doi: 10.3389/fmicb.2024.1413120 (PMC11223650; doi:10.3389/fmicb.2024.1413120)
Supplement: Supplementary file 1 [file Data_Sheet_1.docx]

**Supporting Information**

**Identification and Characterization of a Potential Strain for the Production of Polyhydroxyalkanoate From Glycerol**

Mengheng Xue^1,3,4#^, Rong Huang^2,3,4#^, Wei Liu^1,3,4^, Jian Cheng^3,4^, Yuwan Liu^3,4^, Jie Zhang^3,4,5^, Dingyu Liu^3,4*^, Huifeng Jiang^3,4*^

**Affiliations:**

^1^ School of Life Science and Technology, Wuhan Polytechnic University, Wuhan 430023, China.

^2^ College of Biotechnology, Tianjin University of Science and Technology, Tianjin, 300457, China.

^3^ Tianjin Institute of Industrial Biotechnology, Chinese Academy of Sciences, Tianjin 300308, China.

^4^ National Center of Technology Innovation for Synthetic Biology, Tianjin, 300308, China.

^5^ School of Life Sciences, Division of Life Sciences and Medicine, University of Science and Technology of China, Hefei 230027, China.

^#^These authors contributed equally to this work.

*Corresponding author. Email: Huifeng Jiang, [jiang_hf@tib.cas.cn](mailto:jiang_hf@tib.cas.cn;); Dingyu Liu, liudy@tib.cas.cn.

**Table of content**

**Supplementary Seq. 1**

**Supplementary Fig. 1**

**Supplementary Fig. 2**

**Supplementary Fig. 3**

**Supplementary Fig. 4**

**Table S1**

**Table S2**

**References**

TTTTGCTGTACAGCAAAACTAGCGATTTTTGCTAAACTAACGCCATCCTAGGTACTTTTGCTGTACAGCAAAACTAACGA

Supplementary Seq. 1. A CRISPR sequence predicted by CRISPRFinder on plasmid 2, from 125246 to 125325, totaling 79 bp.


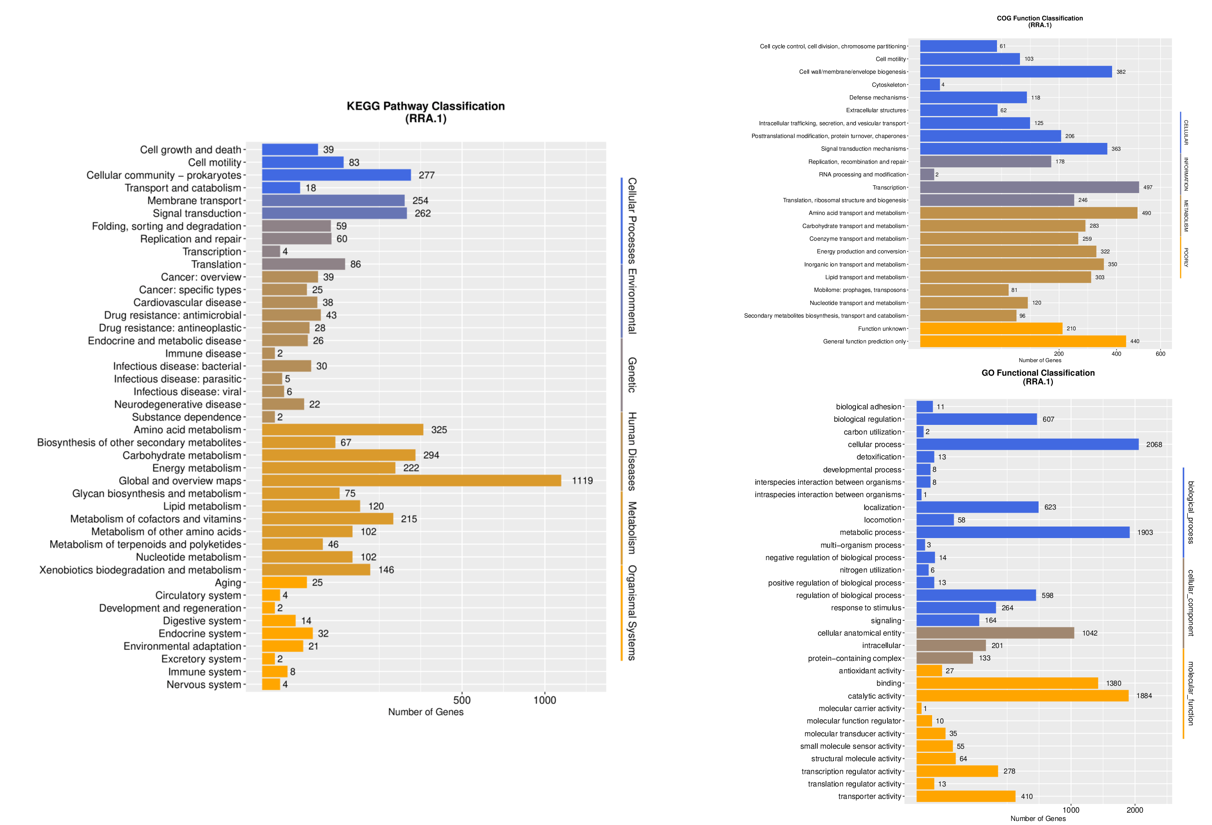


**Supplementary Fig. 1**. The annotations of *RRA.* in KEGG, GO, and COG database.


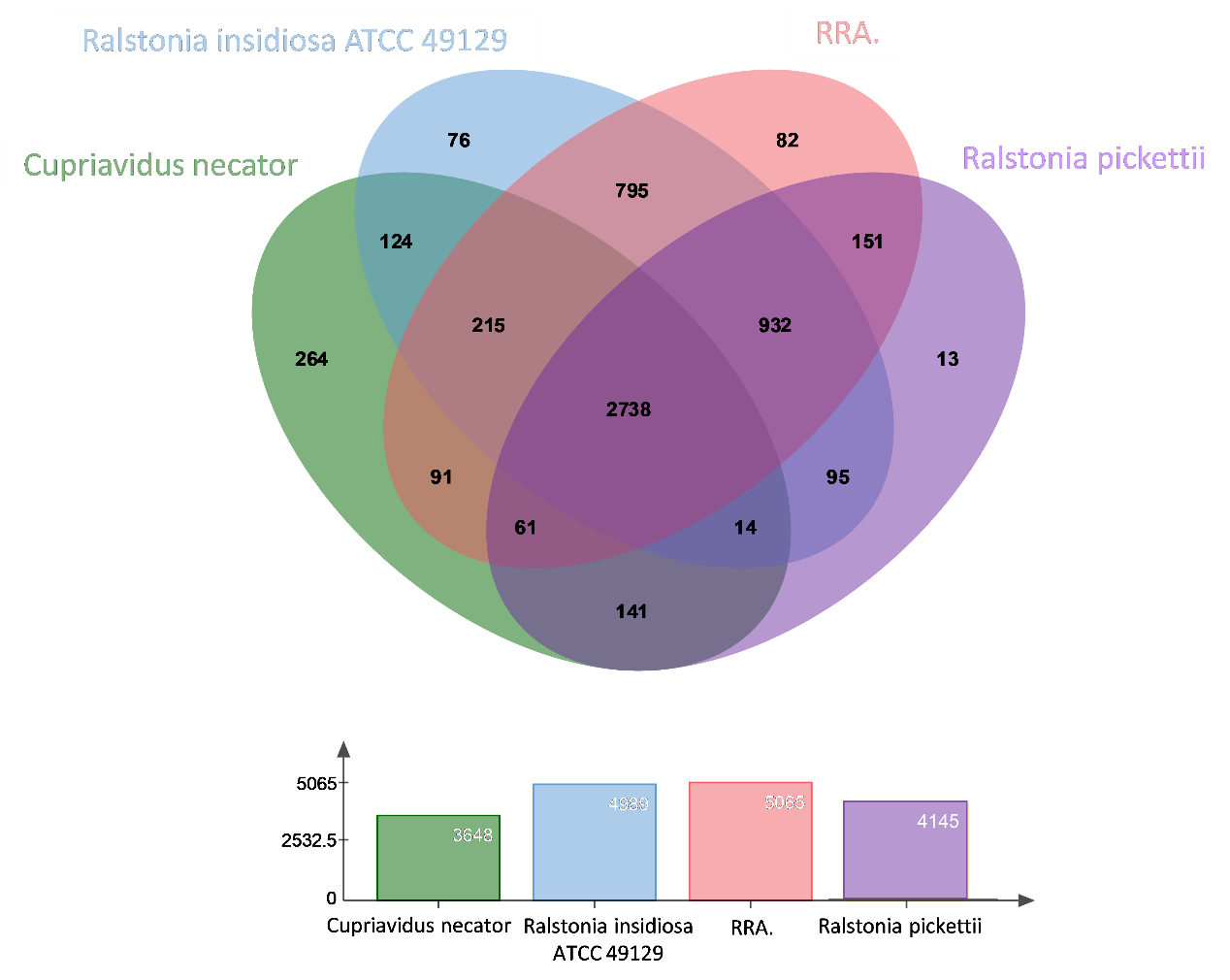


**Supplementary Fig. 2**. Venn diagram displays the distribution of shared orthologous clusters among the *Ralstonia pickettii*, *Cupriavidus necator*, *RRA.* and *Ralstonia insidiosa* ATCC 49129.

A


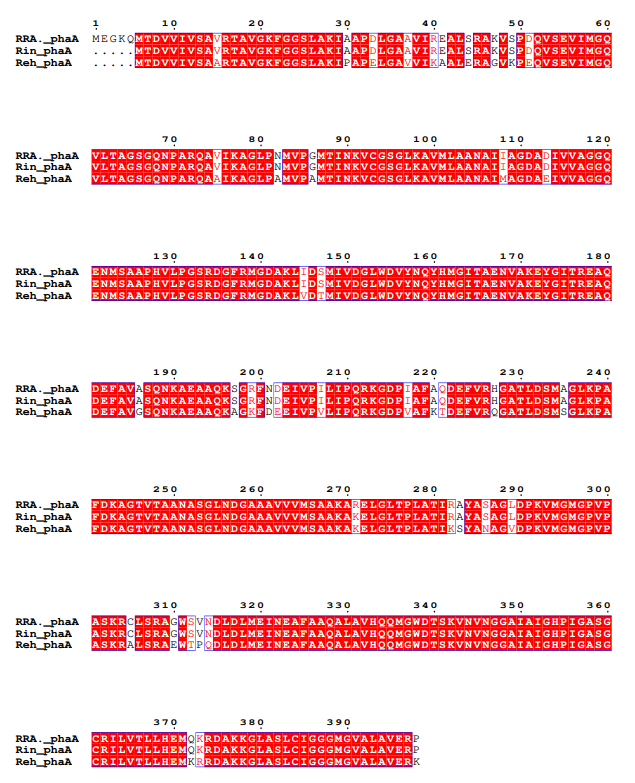


B


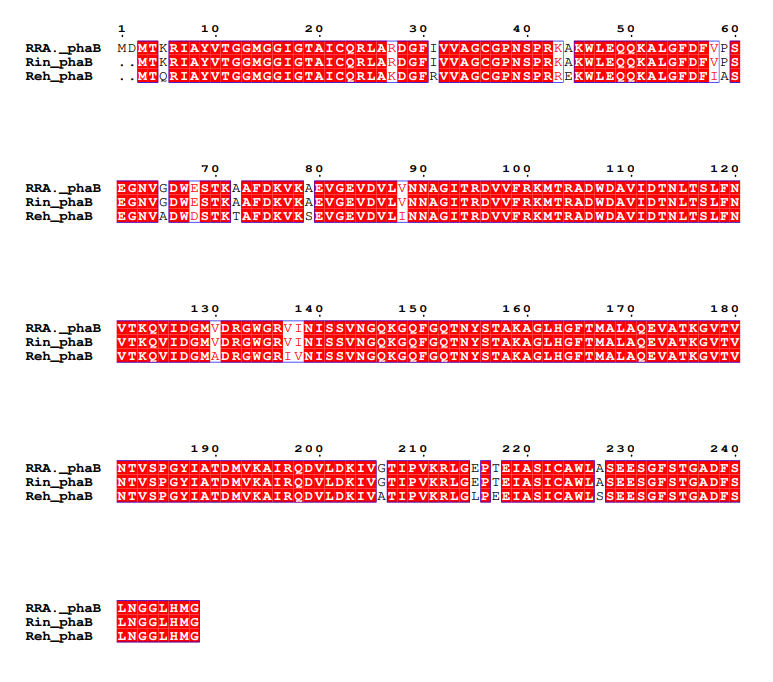


C


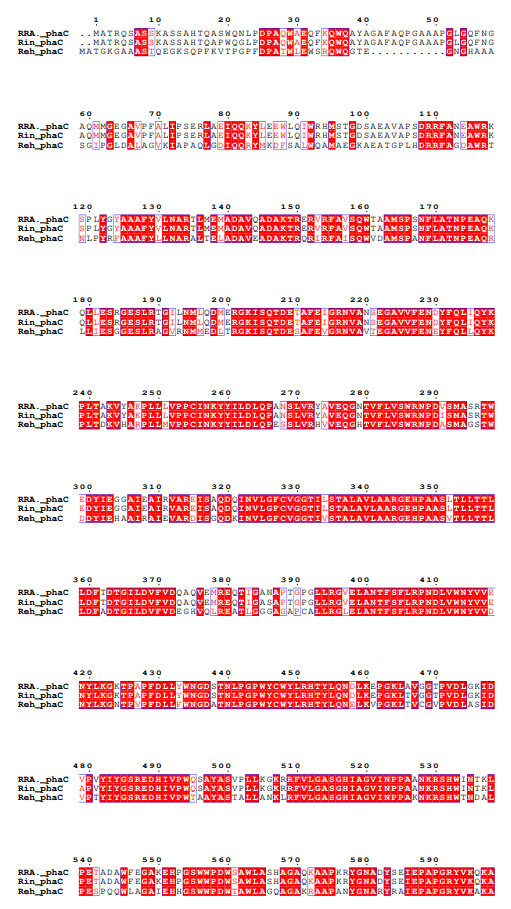


**Supplementary Fig. 3**. Comparison of protein sequences of PhaABC of *RRA.*( RRA.)*, Ralstonia insidiosa* ATCC 49129 (Rin) and *Cupriavidus necator* H16 (Reh). A, Comparison of PhaA, similarity rate is 88.4%. B, Comparison of PhaB, similarity rate is 91.5%. C, Comparison of PhaC, similarity rate is 65.2%.


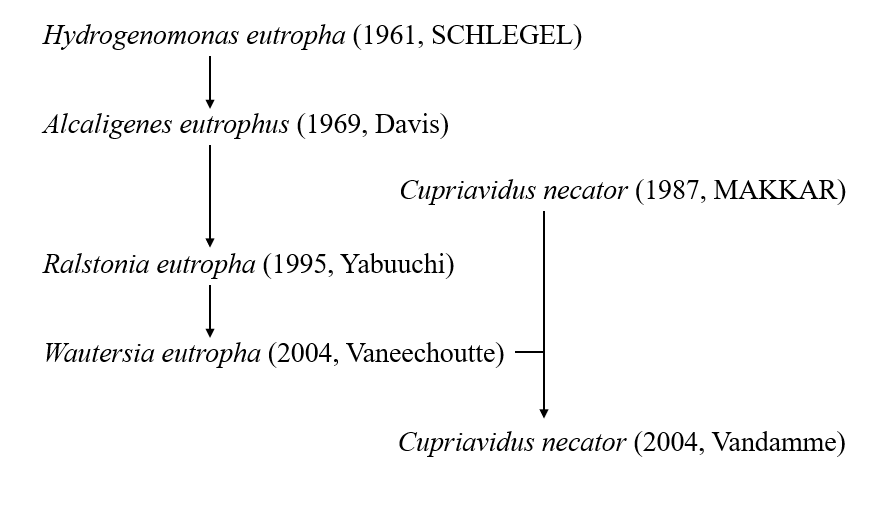


**Supplementary Fig. 4**. *Cupriavidus necator* identification and naming process^[1-6]^.

**Table S1 Strains and plasmids used in this study**

| **Names** | **Descriptions** | **References** |
| --- | --- | --- |
| Strains |  |  |
| *Escherichia coli* BW25113 | F-, DE(araD-araB)567, lacZ4787(del)::rrnB-3, LAM-, rph-1, DE(rhaD-rhaB)568, hsdR514 | [7] |
| *E. coli* DH5α | Rˉ、Mˉ、AMPˉ |  |
| *Ralstonia raxuex* | *Ralstonia raxuex* Wild type, isolated from *R. eutropha* culture | This study |
| Plasmids |  |  |
| pBBR1 | A broad-host-vector that can replicate in *RRA. a*nd *E. coli* BW25113, K^R^ | [8] |
| pBBR1-phaCAB-CE | pBBR1 derivate expressing plasmid, an *RRA.* self contained promoter vector for phaCAB expression, K^R^ | This study |
| pBBR1-phaCAB-IE | pBBR1 derivate expressing plasmid, an L-Arabinose-inducible vector for phaCAB expression, K^R^ | This study |

**Table S2 Primers for DNA cloning in this study**

| **Primers** | **Sequence (5’-3’) a** |
| --- | --- |
| pBBR1-phaCAB-CE |  |
| phaCAB-CE-F | CAGCTGGCAATTCCGACGTCTCCGATTGACAGGCTTGAAACGG |
| phaCAB-CE-R | CAGTCACCTCCTAGCTGACTCAAATCTTACTTCTTCTCAGGCGGCTGGTTG |
| pBBR1-phaCAB-IE |  |
| phaCAB-IE-F | GATCTTTTAAGAAGGAGATATACATATGGCAACGCGTCAATCCGCATCTTC |
| phaCAB-IE-R | CAGTCACCTCCTAGCTGACTCAAATCTTACTTCTTCTCAGGCGGCTGGTTG |
| araC-F | CAGCTGGCAATTCCGACGTCTTATGACAACTTGACGGCTACATCATTCAC |
| araC-R | CTCTGAATGGCGGGAGTATGAAAAGTATGGCTGAAGCGCAAAATGATCC |

**References**

[1] Schlegel, H., G. Gottschalk, and R. Von Bartha, Formation and utilization of poly-β-hydroxybutyric acid by *Knallgas* bacteria (*Hydrogenomonas*). Nature, 1961. 191(4787): p. 463-465.

[2] Davis, D.H., et al., Proposal to reject the genus *Hydrogenomonas*: taxonomic implications. 1969, Microbiology Society. p. 375-390.

[3] Makkar, N. and L. Casida Jr, *Cupriavidus necator* gen. nov., sp. nov.; a nonobligate bacterial predator of bacteria in soil. International journal of systematic and evolutionary microbiology, 1987. 37(4): p. 323-326.

[4] Yabuuchi, E., et al., Transfer of two *Burkholderia* and an *Alcaligenes* species to *Ralstonia* gen. nov.: proposal of *Ralstonia pickettii* (Ralston, Palleroni and Doudoroff 1973) comb. nov., *Ralstonia solanacearum* (Smith 1896) comb. nov. and *Ralstonia eutropha* (Davis 1969) comb. nov. Microbiology and immunology, 1995. 39(11): p. 897-904.

[5] Vandamme, P. and T. Coenye, Taxonomy of the genus *Cupriavidus*: a tale of lost and found. International journal of systematic and evolutionary microbiology, 2004. 54(6): p. 2285-2289.

[6] Vaneechoutte, M., et al., *Wautersia* gen. nov., a novel genus accommodating the phylogenetic lineage including *Ralstonia eutropha* and related species, and proposal of *Ralstonia* [*Pseudomonas*] *syzygii* (Roberts et al. 1990) comb. nov. International journal of systematic and evolutionary microbiology, 2004. 54(2): p. 317-327.

[7] Baba, T., Ara, T., Hasegawa, M., Takai, Y., Okumura, Y., Baba, M., ... & Mori, H. (2006). Construction of Escherichia coli K-12 in-frame, single-gene knockout mutants: the Keio collection. Molecular systems biology, 2(1), 2006-0008.

[8] Kovach, M. E., Elzer, P. H., Hill, D. S., Robertson, G. T., Farris, M. A., Roop II, R. M., & Peterson, K. M. (1995). Four new derivatives of the broad-host-range cloning vector pBBR1MCS, carrying different antibiotic-resistance cassettes. Gene, 166(1), 175-176.
